# Evaluation of Muscle Mass and Quality With an AI‐Based Muscle Ultrasound Imaging System in Patients at Risk of Malnutrition

**DOI:** 10.1002/jcsm.70137

**Published:** 2025-11-25

**Authors:** Juan José López‐Gómez, Lucía Estévez Asensio, Jaime González Gutiérrez, Ángela Cebriá, Olatz Izaola Jauregui, Paloma Pérez López, Emilia Gómez‐Hoyos, David Primo Martín, Rebeca Jiménez Sahagún, Eduardo Jorge Godoy, Daniel A. De Luis Román

**Affiliations:** ^1^ Servicio de Endocrinología y Nutrición Hospital Clínico Universitario de Valladolid Valladolid Spain; ^2^ Centro de Investigación en Endocrinología y Nutrición Universidad de Valladolid Valladolid Spain; ^3^ Health Research Institute of Valladolid (IBioVALL) Valladolid Spain; ^4^ DAWAKO Medtech SL, Parc Cientìfic de la Universitat de Valencia Paterna Spain; ^5^ Técnicas Avanzadas de Desarrollo de Software Centrado en la Persona, Departamento de Informática, Universitat de Valencia Burjassot Spain

**Keywords:** artificial intelligence, disease related malnutrition, muscular ultrasonography, sarcopenia

## Abstract

**Background:**

Sarcopenia is characterized by the loss of muscle mass, quality and function. Ultrasonography provides a non‐invasive method for assessing sarcopenia. Its generalizability remains limited due to certain methodological and population‐specific challenges. This study evaluated the association between AI‐assisted muscle ultrasonography and sarcopenia in patients at risk of malnutrition.

**Methods:**

This observational, cross‐sectional study included 647 patients at risk of malnutrition. Nutritional status was assessed via anthropometry, bioimpedanciometry, quadriceps rectus femoris (QRF) ultrasonography and handgrip strength. An AI‐based imaging system segmented the region of interest (ROI) in transverse QRF images to measure muscle thickness (RFMT), area (RFMA) and pennation angle (RFPA). The Multi‐Otsu algorithm extracted ROI biomarkers: low echogenicity (MiT) and medium echogenicity (FatiT), assumed as a surrogate of muscle and fat percentage of the ROI. Sarcopenia was diagnosed using European Working Group on Sarcopenia in Older People (EWGSOP2) criteria and malnutrition was assessed with Global Leadership Initiative on Malnutrition (GLIM) criteria.

**Results:**

Most of the patients of the study were female (54.4%) and the mean age was 64.83 ± 15.79 years. Malnutrition was present in 530 patients (81.9%) and sarcopenia in 167 patients (25.8%) Among patients with sarcopenia 57.2% had low muscle mass, and 44% had low handgrip strength. Patients with sarcopenia had significantly lower values of RFMT (sarcopenia: 0.89 ± 0.27 cm; no sarcopenia: 1.03 + 0.29 cm; *p* < 0.01) and RFMA (sarcopenia: 2.77 + 1.02 cm²; no sarcopenia: 3.25 + 1.17 cm²; *p* < 0.01). In terms of muscle quality by AI‐assisted ultrasonography, we observed lower values of pennation angle (sarcopenia: 4.97 ± 2.91°; no sarcopenia: 5.50 ± 2.78°; *p* < 0.01), low echogenicity (MiT) (sarcopenia: 45 ± 10.80%; no sarcopenia: 47.39 ± 10.91%; *p* = 0.02) and a higher high echogenicity percentage (NMNFiT) (sarcopenia: 14.99 ± 5.52%; no sarcopenia: 14.76 ± 5.17%; *p* = 0.02). Multivariate analysis showed male sex as a risk factor for sarcopenia (OR = 1.85 (IC 95%: 1.23–2.77); *p* < 0.01), while higher RFMT was protective (OR: 0.18 (IC 95%: 0.04–0.86); *p* = 0.03). For low handgrip strength, higher MiT was protective (OR: 0.07 (IC 95%: 0.13–0.43); *p* < 0.01) after adjusting for age and sex.

**Conclusions:**

In patients at risk of malnutrition, sarcopenia and dynapenia were associated with reduced muscle mass and quality. AI‐based ultrasound parameters, particularly RFMT and MiT, were significantly lower in individuals with sarcopenia and correlated with poorer muscle function, independent of age and sex.

## 1 Background

Sarcopenia is a progressive and generalized skeletal muscle disorder that involves the accelerated loss of muscle mass and function. This age‐related decrease in muscle health can develop a pathology associated with outcomes such as falls, functional decline, frailty and mortality [1]. Although sarcopenia is a pathology classically related to age, some chronic and acute comorbidities can lead to the development of this disease in younger people. Secondary sarcopenia (not age‐related) may occur in systemic diseases, especially in patients with inflammatory processes [2]. The mechanisms of sarcopenia development share multiple pathways with another disease such as disease‐related malnutrition (DRM); for this reason these two entities are usually related either in patients of older age or those of younger age [3].

The diagnosis of sarcopenia is based on a decrease in muscle mass, muscle function and functional decline. Evaluating muscle function is relatively straightforward, as we have several validated tools to assess this condition [4]. However, measuring muscle mass is more challenging for several reasons: gold‐standard techniques like computed tomography (CT) or dual‐energy X‐ray absorptiometry (DEXA) are not always available in all centers and require patients to undergo additional testing; bedside techniques like anthropometry or bioimpedance analysis can be affected by factors such as body water or obesity, leading to inaccuracies in muscle estimation [5]. On the other hand, muscle quality is a key characteristic included in the definition of sarcopenia but is not well assessed due to the difficulty in measuring and standardizing it. This muscle parameter is associated with the progression of sarcopenia and the prognosis of patient comorbidities [6].

Muscle ultrasonography is a useful tool in nutritional assessment, particularly for diagnosing malnutrition and sarcopenia as indicators of low muscle mass. This technique also enables the evaluation of patient prognosis in various conditions associated with disease‐related malnutrition and sarcopenia. For instance, Fernández‐Jiménez et al. demonstrated that quadriceps rectus femoris (QRF) ultrasonography serves as a reliable prognostic marker for 12‐month mortality in patients with pulmonary fibrosis [7]. Similarly, García‐García et al. in the AnyVida Trial, revealed that QRF thickness is a predictor of mortality in cancer patients [8]. Moreover, the lowest quartiles of quadriceps thickness have been shown to be a risk factor for readmission in patients with amyotrophic lateral sclerosis [9]. In addition, muscle ultrasonography can be employed to monitor medical nutritional therapy in both hospitalized and community‐based patients. Various types of oral nutritional supplements have been evaluated using muscle ultrasonography, with mixed results. For example, Herrera et al. found no significant changes in QRF ultrasonography among patients with cancer‐induced sarcopenia [10]. However, other studies have reported distinct changes in ultrasound evaluations depending on the type of oral nutritional supplement used. Our research group, for instance, observed an increase in muscle thickness in patients with disease‐related malnutrition who were treated with either an energy‐dense high protein formula or an oral nutritional supplement enriched with β‐hydroxybutyrate [11, 12].

Muscle ultrasonography is a technique that allows for the evaluation of both muscle mass and muscle quality. Several muscles have been used to assess sarcopenia and nutritional status, but the most frequently examined muscle is the quadriceps, specifically the components: rectus femoris and the vastus intermedius [13]. Muscle mass, particularly the thickness and area of the QRF, has become a primary focus for diagnosing sarcopenia. For instance, the DRECO study defined specific thresholds for diagnosing sarcopenia based on these two parameters. Additionally, muscular ultrasonography serves not only as a technique for quantifying muscle mass but also as an effective approach for assessing muscle quality. This evaluation is conducted by analyzing echogenicity and muscle architecture, which are linked to inflammation and decreased strength [14].

Muscle quality assessed by ultrasonography is based on the evaluation of muscle composition through mean pixel intensity (brightness) within a region of interest (ROI). This method aims to differentiate between contractile and non‐contractile elements, such as fibrous tissue and fat tissue [6]. Assessing muscle quality can provide insight into muscle deterioration before observable reductions in mass or functional impairments occur. Several studies have investigated this condition, identifying a relationship between muscle characteristics and function. For instance, muscle echo‐intensity has demonstrated comparable correlations with metabolic parameters and physical performance metrics, including isokinetic knee extension strength and handgrip strength, when compared to muscle quality assessed via computed tomography (CT) [15]. Furthermore, the combination of lean soft tissue measurements from DEXA and echointensity has shown better predictive accuracy for muscle strength than lean soft tissue alone, as reported in a predictive model developed by Bourgeois et al. [16].

Ultrasound image analysis systems with artificial intelligence (AI) enable the automatic segmentation of regions of interest (ROIs), facilitating the interpretation of images, reducing processing time and minimizing interobserver variability to standardize muscle mass measurements [17, 18]. Additionally, the use of these tools, particularly through histogram‐based algorithms, allows for the assessment of muscle quality and the differentiation of ROI components (muscle mass, fat mass and other structures, including fibrosis) based on echogenicity variations, using adapted thresholds specific to each image [11, 12]. The use of AI for image segmentation in patients with disease‐related malnutrition has proven to be at least as effective as human observers in some studies. García‐Herreros et al. demonstrated that automatic segmentation performed by an AI tool (PIIXMED) achieved an intraclass correlation coefficient of 0.912 for subcutaneous fat, 0.96 for muscle thickness and 0.99 for muscle area [17]. On the other hand, muscle quality characteristics measured using AI technology have been associated with inflammation in patients at risk of malnutrition, showing a lower muscle percentage and higher fat content in the region of interest (ROI) among those with high levels of inflammation [19].

The use of AI‐based tools for analysing muscular ultrasonography images facilitates the early diagnosis of conditions such as sarcopenia and supports the identification of new biomarkers, which may guide the development of targeted therapies. However, their application requires further evaluation in populations with various pathologies to assess the relationship with clinical and functional parameters of muscle health. The aim of this study is to assess differences in muscle mass and quality parameters, obtained through an AI‐based muscle ultrasound imaging system, between patients with and without sarcopenia, and to evaluate their association with the diagnosis of sarcopenia and its components (low muscle mass and dynapenia) in patients at risk of malnutrition.

## 2 Methods

### 2.1 Study Design and Eligibility Criteria

This cross‐sectional observational study included patients over the age of 18 who were at risk of malnutrition. These participants were recruited from the Endocrinology and Nutrition Service of the University Clinic Hospital of Valladolid, Spain, between January 2021 and September 2024. Exclusion criteria include stage IV or higher chronic kidney disease, uncontrolled liver disease, terminal oncologic conditions and refusal to sign the informed consent.

Participants underwent assessment that included a nutritional history, anthropometric measurements, electrical bioimpedance analysis, quadriceps rectus femoris muscle ultrasonography and handgrip strength tests.

The study was approved by the Medical Research Ethics Committee (CEIm) of the East Valladolid Area (code: PI 20‐1886 and PI 23‐341). All study procedures complied with the principles of the Declaration of Helsinki. Signed informed consent was obtained from all eligible participants prior to enrolment.

### 2.2 Variables

#### 2.2.1 Anthropometric Measures

The anthropometric variables measured were current body weight (kg), considered to be the weight recorded at the time of clinical evaluation; usual body weight (kg), considered to be the patient's usual weight during the months prior to the onset of the pathological condition that triggered malnutrition, This value was obtained through clinical interviews and review of medical records when available; height (m); body mass index [current weight/height × height (kg/m^2^), arm circumference (cm); calf circumference (cm)]; and percentage of body weight loss (usual weight‐current weight/usual weight × 100).

#### 2.2.2 Electrical Bioimpedanciometry (BIA)

This method involved using a bioimpedance analyser (BIA 101 Anniversary; EFG Akern, Pisa, Italy). The BIA measurements were taken between 8:00 and 10:00 AM after an overnight fast and following 15 min in a supine position. The raw electrical data collected included reactance (ohms), resistance (ohms) and phase angle (degrees). The appendicular skeletal muscle index (ASMI), used to diagnose low muscle mass and malnutrition, was estimated using Sergi's Formula [20].

#### 2.2.3 AI‐Based Muscular Ultrasonography

Ultrasonographic examination of the quadriceps rectus femoris (QRF) muscle was performed on the dominant lower limb using a 10 MHz probe with a multifrequency 7L4P linearprobe in MSK (musculoskeletal) mode (Mindray Z60, Madrid, Spain). The measurements were taken with the patient lying in a supine position, with the probe positioned perpendicular to the muscle in the transverse axis of the dominant leg (the lower third of the distance between the iliac crest and the upper border of the patella) [13]. Ultrasound examinations were performed by trained personnel, following a standardized protocol (the following settings were applied consistently across all patients to ensure standardization: frequency 10 MHz; depth 4.5–4.7 cm; gain 43–45; frame rate 23 frames per second; dynamic range 155). All operators followed a standardized protocol to ensure consistency in image acquisition. All individuals involved received specific training focused on muscle imaging techniques, including probe positioning, image optimization and minimizing tissue compression to avoid distortion. Minimal compression was applied to the limb during imaging, just enough to obtain a clear image without distorting the subcutaneous tissue or underlying muscle structure. This approach was intended to preserve anatomical accuracy and consistency across patients. However, no specific methods were used to quantitatively monitor or measure the level of compression applied to the muscle during imaging. For each patient, three transverse images of the rectus femoris muscle were captured, and the image with the highest quality, based on clarity and anatomical definition, was selected for analysis. All images were saved in JPEG format, and although this format involves lossy compression, care was taken to ensure minimal loss of diagnostic information.

**FIGURE 1:**
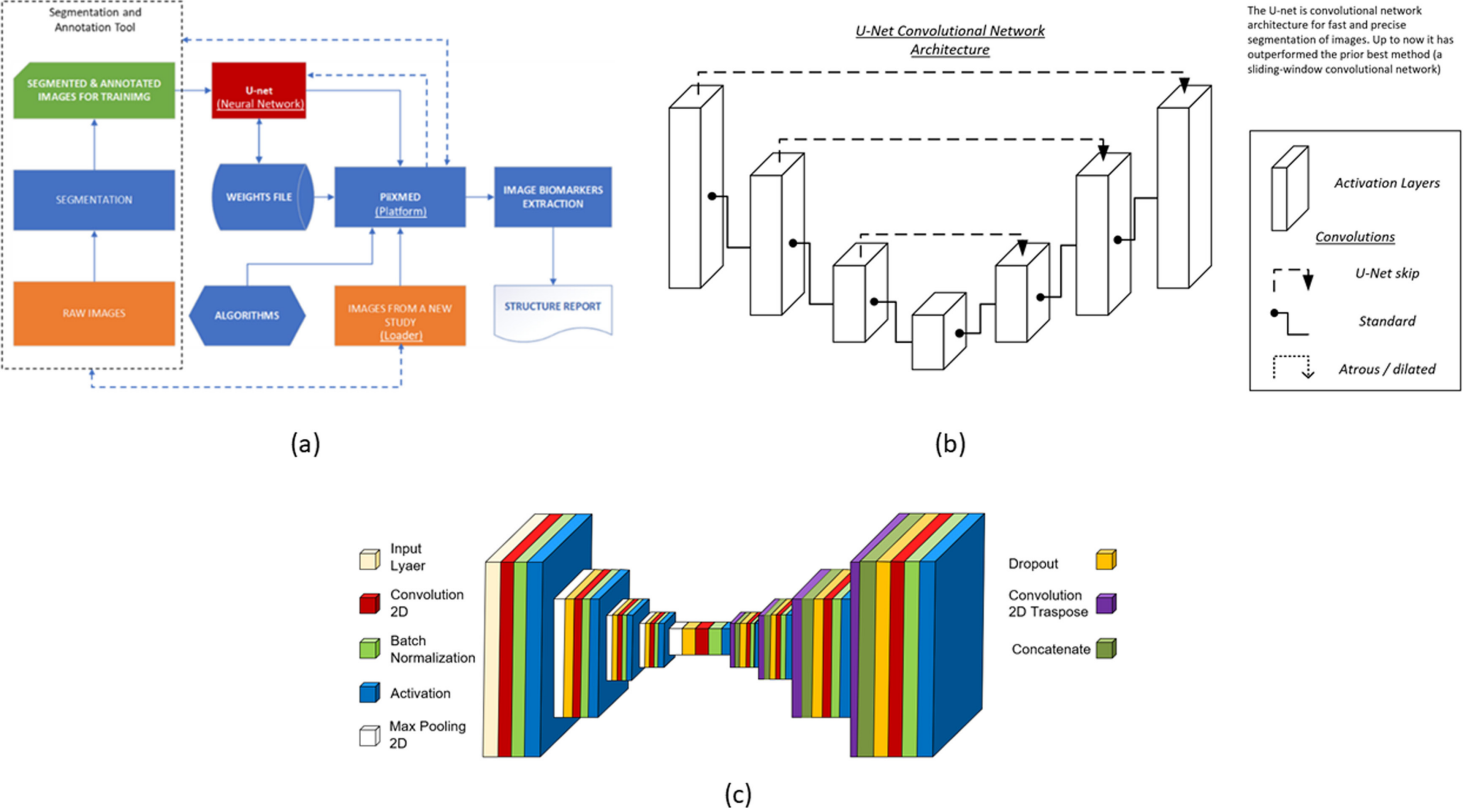
Convolutional neural networks applied in PIIXMED. (a) Deep learning convolutional network workflow for U‐net‐based image analysis; (b) U‐net convolutional network architecture; (c) convolutional network and their different layers.

The images obtained from the ultrasonography were processed using an AI‐based ultrasound imaging system (PIIXMED; DAWAKO MedTech; Valencia, Spain) (Figure 1). The PIIXMED system for automatic segmentation and analysis of medical ultrasound images is a cloud‐based diagnostic support tool. It utilizes a convolutional neural network (CNN) with a U‐Net architecture, originally developed by the University of Freiburg [21]. This architecture is optimized to perform accurate segmentation with a limited number of training images. The PIIXMED system allows for 2D feature extraction in conventional B‐Mode ultrasound imaging and can calculate single values per feature for a region of interest (ROI). The system integrates radiomics‐based algorithms using an open‐source Python package [22], enabling the extraction of quantitative features such as anatomical measurements, echogenicity, texture and fractal dimension. These features are used to derive surrogate biomarkers related to muscle mass and quality. Various biomarkers were extracted and processed by analyzing the identified features and applying various algorithms to assess the ROI's morphological architecture, muscle quality based on echogenicity, and different texture‐based biomarkers. The biomarkers obtained and analyzed are further developed later.

Validation of the segmentation performance has been previously reported by García‐Herreros et al., showing high intraclass correlation coefficients when compared to human observers: 0.912 for subcutaneous fat, 0.96 for muscle thickness and 0.99 for muscle area [17]. The evaluated muscle mass parameters included the rectus femoris muscle area (RFMA) in cm^2^ and the rectus femoris muscle thickness (RFMT) in cm, representing the cross‐sectional muscle area and thickness within the ROI of the muscle belly in transverse section. Subcutaneous fat thickness (SFT) was measured to determine the thickness of subcutaneous adipose tissue in the longitudinal section.

**FIGURE 2:**
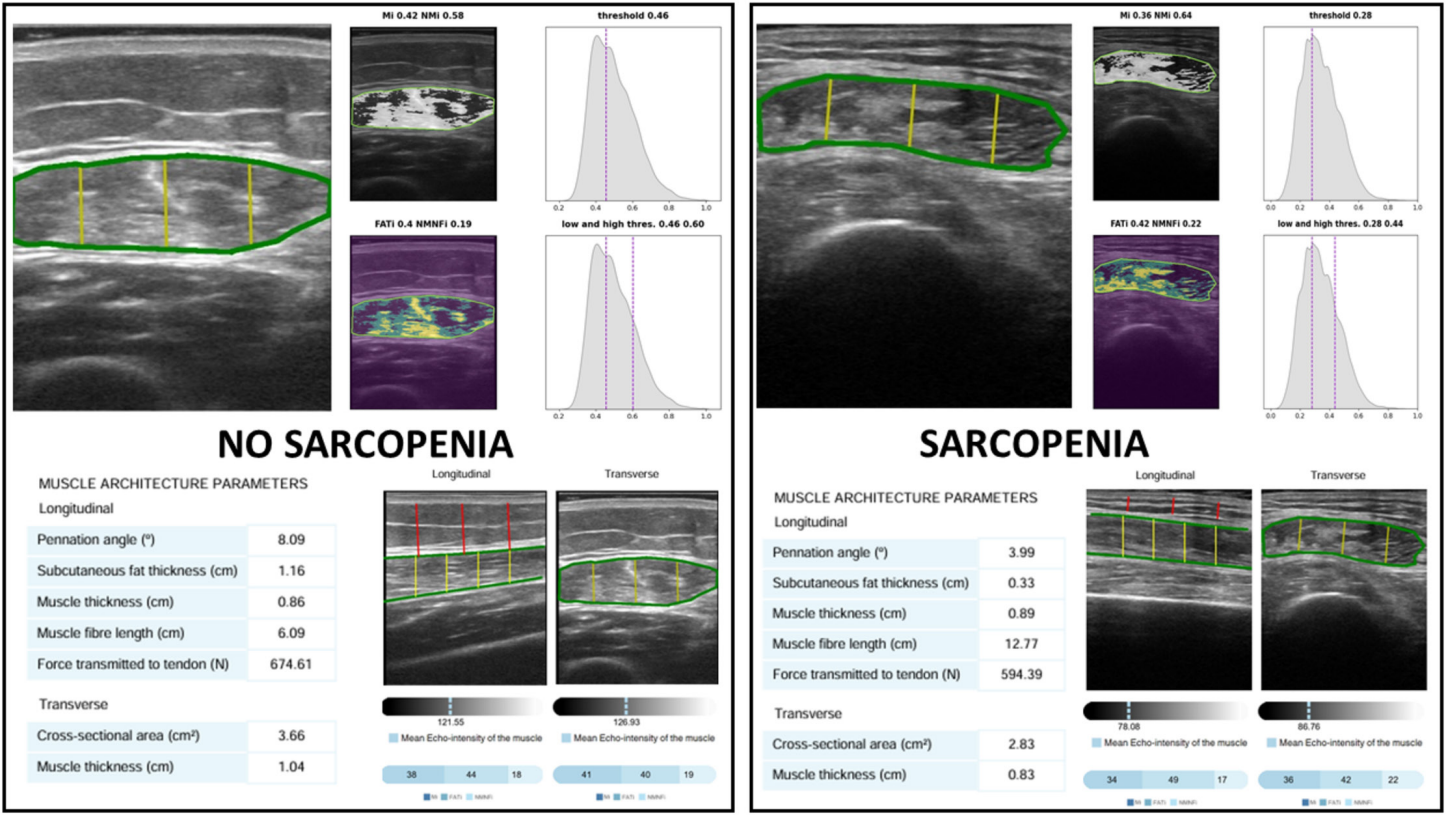
Image analysis by PIIXMED in a patient with sarcopenia and other without sarcopenia. Upper images: cross‐sectional quadriceps rectus femoris ultrasonography image from two patients: one diagnosed with sarcopenia and one without, based on EWGSOP2 criteria. The images were analysed using artificial intelligence, including histograms and thresholds derived from the multi‐Otsu algorithm. The pixels are differenced as low echogenicity (purple), medium echogenicity (green) and high echogenicity (yellow); Lower images: Output of the PIIXMED tool with numeric measures and the muscle echogenicity percentage of Region of Interest in the bottom bar low echogenicity (Mi), medium echogenicity (FATi), high echogenicity (NMNFi).

Muscle quality was assessed by measuring the pennation angle in degrees in the longitudinal section, which is the angle between the muscle fibres and the lower aponeurosis (a higher pennation angle indicates a greater ability for muscle strength). Muscle quality indexes were determined using a multithresholding algorithm based on histogram echogenicity and grey intensity, defining thresholds to separate ultrasound image pixels into different classes. The Multi‐Otsu algorithm builds upon the traditional Otsu method, which is commonly used for segmenting images based on pixel intensity. While the original Otsu technique separates an image into two distinct regions, typically foreground and background, Multi‐Otsu extends this approach by dividing the image into three or more intensity‐based classes. The multi‐Otsu thresholding algorithm is a histogram‐based method applied to image echogenicity (grey‐level intensity). It determines a predefined number of thresholds that partition the pixels of an input image—here, ultrasound images—into distinct classes based on grey‐level distributions [23]. This is particularly useful for images that contain multiple areas of interest. The algorithm determines the optimal threshold values by minimizing the variance within each class and maximizing the variance between classes. This process relies on statistical analysis of the image's histogram, allowing for more refined segmentation across varying intensity levels. In this context of muscle ultrasound, the multi‐Otsu algorithm is used to classify tissue regions based on their echogenicity (brightness in the image), which may reflect different components such as: muscle mass (low echogenicity), adipose tissue (medium echogenicity) and areas without muscle or fat (high echogenicity or artefacts). This algorithm calculates threshold values for three echogenicity‐based categories in the transverse image, low (MiT), medium (FATiT) and high (NMNFiT), each expressed as a percentage of the region of interest (ROI). In practice, the multi‐Otsu algorithm computes thresholds according to the number of classes specified. By default, it generates three classes, which correspond to two thresholds values. These thresholds are typically visualized as vertical violet lines on the histogram (Figure 2). The resulting indices are reported as percentages of the ROI [23]. The AI tool provides an output displaying the ROI segmentation, muscle mass values and muscle quality values, expressed as the percentage of distinct echogenicity areas within the ROI (Figure 2). Although the tool is capable of analysing both longitudinal and transverse quality indices, this study focused on transverse indices, as they are more widely supported by existing experience and literature, and refer to a region that is easier to standardize, with a lower percentage of interobserver variability.

#### 2.2.4 Muscle Strength

Muscle functionality was assessed using handgrip strength measured by a JAMAR dynamometer (Basel, Switzerland). The test was made with patients seated with their dominant arm at a right angle to the forearm and performing handgrip.

#### 2.2.5 Nutritional Diagnosis

○ Malnutrition Diagnosis: The diagnosis of malnutrition was conducted using the Global Leadership Initiative on Malnutrition (GLIM) criteria. Patients must have one phenotypic criterion and one etiologic criterion [24].
○ Sarcopenia Diagnosis: The diagnosis of sarcopenia was made with the European Working Group of Sarcopenia in Older People (EWGSOP2) criteria [2]. The patients must have an altered handgrip strength (Low muscle strength was defined as < 27 kg in men and < 16 kg in women) and a low muscle mass (low muscle mass was defined as ASMI < 7 kg/m^2^ in men and ASMI < 5.5 kg/m^2^ determines by BIA). Patients with altered handgrip strength with no low muscle mass were considered as probable sarcopenia or dynapenia.
○ AI based Muscle Ultrasonography Diagnosis System: To evaluate the impact of muscle parameters on the diagnosis of sarcopenia and dinapenia, we employed an integrative approach of different muscle US variables was employed to diagnose Low muscle Mass and Low Muscle Quality using the median values from our sample. In this analysis, one point was assigned to every altered component of muscle mass and quality:
  - Muscle mass score: low muscle mass was defined as a low RFMA (men < 3.48 cm^2^; women < 2.62 cm^2^; 1 point), a low RFMT (men < 0.89 cm; women < 1.06 cm; 1 point), or both (2 points).
  - Muscle quality score: low muscle quality was defined as low MiT (men < 45.88%; women < 43.92%; 1 point); high FATiT (men > 39.41%; women > 40.27%; 1 point); or low pennation angle (men < 5.76°; women < 4.78°; 1 point). The score was a result of the sum of the three parameters (0–3 points).

### 2.3 Data Analysis

Statistical analysis was conducted using the SPSS 15.0 software package (SPSS Inc., Chicago IL, USA), officially licensed by the University of Valladolid. A normality test for continuous variables was performed using the Kolmogorov–Smirnov test. Normally distributed continuous variables are expressed as mean (standard deviation) and non‐normally distributed continuous variables are expressed as median (interquartile range). Qualitative variables are represented by the number and percentage of the total sample.

Differences between parametric continuous variables were analyzed using the unpaired Student's *t*‐test, while differences between non‐parametric variables were analyzed using the Mann–Whitney *U*‐test. If comparisons among more than two groups were necessary, the ANOVA test (with the Bonferroni post hoc test) was employed. Correlation analysis was performed to evaluate the relationship between quantitative variables.

A binary logistic regression was conducted in a multivariate analysis to assess the relationship of the variables with the prognosis. In the multivariate analysis, two models were considered for the analysis of the probability of development of sarcopenia and dynapenia:

- Model 1: It was an adjusted model with absolute values of quantitative variables age, RFMA, RFMT and MiT; and qualitative variable as gender.
- Model 2: It was an adjusted model with absolute values of age; and qualitative values of gender and the muscle mass and muscle quality score.
*p*‐value under 0.05 was consider significative.

## 3 Results

### 3.1 Sample Description

Six hundred forty‐seven patients at risk of malnutrition were recruited in this study. Three hundred fifty‐two patients (54.4%) were women. The average age of patients was 64.83 + 15.79 years; 268 patients (41.4%) had more than 70 years.

The main pathologies that lead to the risk of malnutrition were oncologic diseases 321 patients (49.6%); neurologic diseases 113 patients (17.5%); gastroenterological diseases 66 patients (10.2%); and cardiopulmonary diseases 61 patients (9.5%).

The nutritional diseases that patients suffered were Malnutrition 530 (81.9%) patients; sarcopenia 167 (25.8%) patients [low handgrip strength (dynapenia): 285 (44%) patients; low muscle mass (assessed by BIA): 370 (57.2%) patients].

Body composition variables showed higher anthropometric variables, higher BIA variables, higher values of muscle mass and quality in men than women assessed by AI‐assisted muscular ultrasonography and higher handgrip strength (Table 1).

**TABLE 1:** Differences in body composition variables between sex.

	Total (*n* = 647)	Men (*n* = 295)	Women (*n* = 352)	*p*
Age (years)	64.83 ± 15.79	66.44 ± 14.47	63.48 ± 16.72	0.02
Anthropometry				
BMI (kg/m^2^)	22.61 ± 4.94	23.69 ± 4.41	21.70 ± 5.17	< 0.01
Arm circumference (cm)	24.64 ± 3.69	25.63 ± 3.18	23.75 ± 3.89	< 0.01
Calf circumference (cm)	31.91 ± 4.05	32.71 ± 4.09	31.24 ± 3.91	< 0.01
Bioimpedanciometry				
Resistance (ohm)	590.28 ± 112.95	539.01 ± 94.69	633.53 ± 109.05	< 0.01
Reactance (ohm)	50.92 ± 12.23	48.54 ± 11.21	52.92 ± 12.70	< 0.01
Phase angle (°)	4.96 ± 0.97	5.16 ± 0.98	4.79 ± 0.93	< 0.01
ASMI (kg/m^2^)	6.11 ± 2.82	6.82 ± 1.42	5.51 ± 0.86	< 0.01
Rectus femoris muscular ultrasonography				
SFT (cm)	0.77 ± 0.45	0.53 ± 0.27	0.98 ± 0.47	< 0.01
RFMT (cm)	0.98 ± 0.29	1.08 ± 0.31	0.90 ± 0.26	< 0.01
RFMA (cm^2^)	3.10 ± 1.15	3.56 ± 1.21	2.72 ± 0.94	< 0.01
RFMAI (cm^2^/m^2^)	1.18 ± 0.41	1.27 ± 0.43	1.09 ± 0.38	< 0.01
MiT (%)	46.55 ± 10.79	47.98 ± 11.66	45.35 ± 9.85	< 0.01
FATiT (%)	39.29 ± 7.13	38.78 ± 7.81	39.71 ± 6.49	0.09
NMNFiT (%)	14.16 ± 5.23	13.24 ± 5.26	14.93 ± 5.08	< 0.01
Pennation angle (°)	5.32 ± 2.82	5.79 ± 2.89	4.90 ± 2.69	< 0.01
Muscle function				
Handgrip strength (kg)	21.81 ± 9.34	26.70 ± 9.13	17.71 ± 7.34	< 0.01

Abbreviations: ASMI: appendicular skeletal muscle index; BMI: body mass index; FATiT: medium echogenicity percentage; MiT: low echogenicity percentage; NMNFiT: high echogenicity percentage; RFMA: rectus femoris muscle area; RFMAI: rectus femoris muscle area index; RFMT: rectus femoris muscle thickness; SFT: subcutaneous fat thickness.

### 3.2 AI‐Assisted Muscular Ultrasonography in Sarcopenia and Dynapenia

In cross‐sectional view of ultrasonography, 569 patients (87.9%) showed complete capture of the quadriceps rectus femoris, while 78 patients (12.1%) had incomplete capture of this muscle. There was no significant difference in rectus femoris area capture between patients with sarcopenia and those without (sarcopenia: 16 (8.9%) patients; no sarcopenia: 62 (13.3%) patients; *p* = 0.13).

#### 3.2.1 Sarcopenia

Patients with sarcopenia had worse values of muscle mass parameters (Table 2). These differences were maintained when we stratified by sex (Table 2).

**TABLE 2:** Differences in muscle ultrasonography depending on dynapenia and sarcopenia diagnosis in total sample and stratified by sex.

	Overall					
	Sarcopenia			Dynapenia		
	Sarcopenia (*N* = 167)	No sarcopenia (*N* = 448)	*p*	Dynapenia (*N* = 285)	No dynapenia (*N* = 339)	*p*
SFT (cm)	0.66 ± 0.35	0.79 ± 0.47	0.02	0.80 ± 0.48	0.72 ± 0.41	0.02
RFMT (cm)	0.88 ± 0.27	1.03 ± 0.29	< 0.01	0.93 ± 0.29	1.03 ± 0.29	< 0.01
RFMA (cm^2^)	2.77 ± 1.02	3.25 ± 1.17	< 0.01	2.91 ± 1.11	3.28 ± 1.16	< 0.01
RFMAI (cm^2^/m^2^)	1.05 ± 0.36	1.23 ± 0.42	< 0.01	1.13 ± 0.41	1.23 ± 0.41	< 0.01
MiT (%)	44.99 ± 10.80	47.38 ± 10.91	0.02	44.46 ± 10.44	48.61 ± 10.91	< 0.01
FATiT (%)	40.02 ± 7.00	38.86 ± 7.29	0.08	40.55 ± 6.83	38.04 ± 7.31	< 0.01
NMNFiT (%)	14.99 ± 5.52	13.76 ± 5.17	0.01	14.98 ± 5.39	13.35 ± 5.05	< 0.01
Pennation Angle (°)	4.97 ± 2.91	5.50 ± 2.78	0.02	5.05 ± 2.81	5.57 ± 2.82	0.02

	Men			Women		
	Dynapenia (*N* = 137)	No dynapenia (*N* = 147)	*p*‐value	Dynapenia (*N* = 148)	No dynapenia (*N* = 192)	*p*‐value
SFT (cm)	0.57 ± 0.29	0.48 ± 0.24	0.02	1.04 ± 0.51	0.92 ± 0.42	0.02
RFMT (cm)	1.04 ± 0.31	1.12 ± 0.30	< 0.01	0.84 ± 2.39	0.96 ± 0.26	< 0.01
RFMA (cm^2^)	3.38 ± 1.18	3.75 ± 1.24	< 0.01	2.48 ± 0.86	2.92 ± 0.95	< 0.01
RFMAI (cm^2^/m^2^)	1.23 ± 0.42	1.32 ± 0.44	< 0.01	1.04 ± 0.38	1.15 ± 0.37	< 0.01
MiT (%)	46.11 ± 11.11	50.10 ± 12.04	< 0.01	42.94 ± 9.51	47.46 ± 9.84	< 0.01
FATiT (%)	39.92 ± 7.30	37.50 ± 8.23	< 0.01	41.14 ± 6.34	38.46 ± 6.50	< 0.01
NMNFiT (%)	14.0 ± 5.45	12.0 ± 5.06	< 0.01	15.92 ± 51.91	14.07 ± 4.93	< 0.01
Pennation Angle (°)	5.62 ± 3.06	5.97 ± 2.72	0.02	4.50 ± 2.43	5.25 ± 2.87	0.02

	MEN			WOMEN		
	SARCOPENIA N= 85	NO SARCOPENIA N= 196	p‐value	SARCOPENIA N= 82	NO SARCOPENIA N= 252	p‐value
SFT (cm)	0.50 +2.72	0.52+2.69	0.59	0.83 +0.35	1.02 +0.49	<0.01
RFMT (cm)	0.98 +2.81	1.13+0.31	<0.01	0.79+0.22	1.16+0.37	<0.01
RFMA (cm^2^)	3.18+1.05	3.73+1.25	<0.01	2.34+0.79	2.87+0.95	<0.01
RFMAI (cm^2^/m^2^)	1.15+0.36	1.33+0.45	<0.01	0.95+0.35	1.16+0.37	<0.01
MiT (%)	46.35+11.33	48.95+11.98	0.09	43.59+10.11	46.17+9.86	0.04
FATiT (%)	39.74+7.57	38.21+8.03	0.14	40.30+6.39	39.36+6.62	0.26
NMNFiT (%)	13.90+52.97	12.83+53.15	0.12	16.11+5.55	14.48+4.95	0.01
Pennation Angle (º)	5.54+3.36	5.92+2.66	0.32	4.38+2.20	5.14+2.83	0.03

Abbreviations: ASMI: appendicular skeletal muscle index; BMI: body mass index; FATiT: medium echogenicity percentage; MiT: low echogenicity percentage; NMNFiT: high echogenicity percentage; RFMA: rectus femoris muscle area; RFMT: rectus femoris muscle thickness; SFT: subcutaneous fat thickness.

In terms of muscle quality by AI‐assisted ultrasonography, we observed lower values of pennation angle and higher values of high echogenicity structures (NMNFiT) (Table 2). There were no differences in medium echogenicity (FATi) percentage. The differences were seen in aggregate and maintained in women when we stratified by sex, but there were no differences in men (Table 2).

#### 3.2.2 Dynapenia

Patients with dynapenia (altered values of handgrip strength with EWGSOP2 criteria) had worse values of muscle mass parameters: RFMT and RFMA (Table 2). These differences were seen in aggregate and maintained for each sex when stratified (Table 2).

In terms of muscle quality by AI‐assisted ultrasonography, we observed lower values of pennation angle; low echogenicity percentage (MiT); medium echogenicity (FATi) and higher values of high echogenicity percentage (NMNFiT) (Table 2). The differences were seen in aggregate and maintained in women when we stratified by sex, but there were no differences in men (Table 2).

### 3.3 Diagnosis of Muscle Mass and Quality by Ultrasonography

They were made two aggregates for muscle mass (Muscle Mass Score) and muscle quality (muscle quality score) based on AI‐assisted ultrasonography; these aggregates were based on the sum of points assigned to poorer muscle mass or quality values, adjusted by sex, as described in Section 2. With respect to muscle mass score, 44% of patients had 0 points, 12% of patients had 1 point and 44% had 2 points. For muscle quality score, 22% of patients had 0 points; 26% of patients had 1 point; 30% of patients had 2 points; and 22% of patients had 3 points.

**FIGURE 3:**
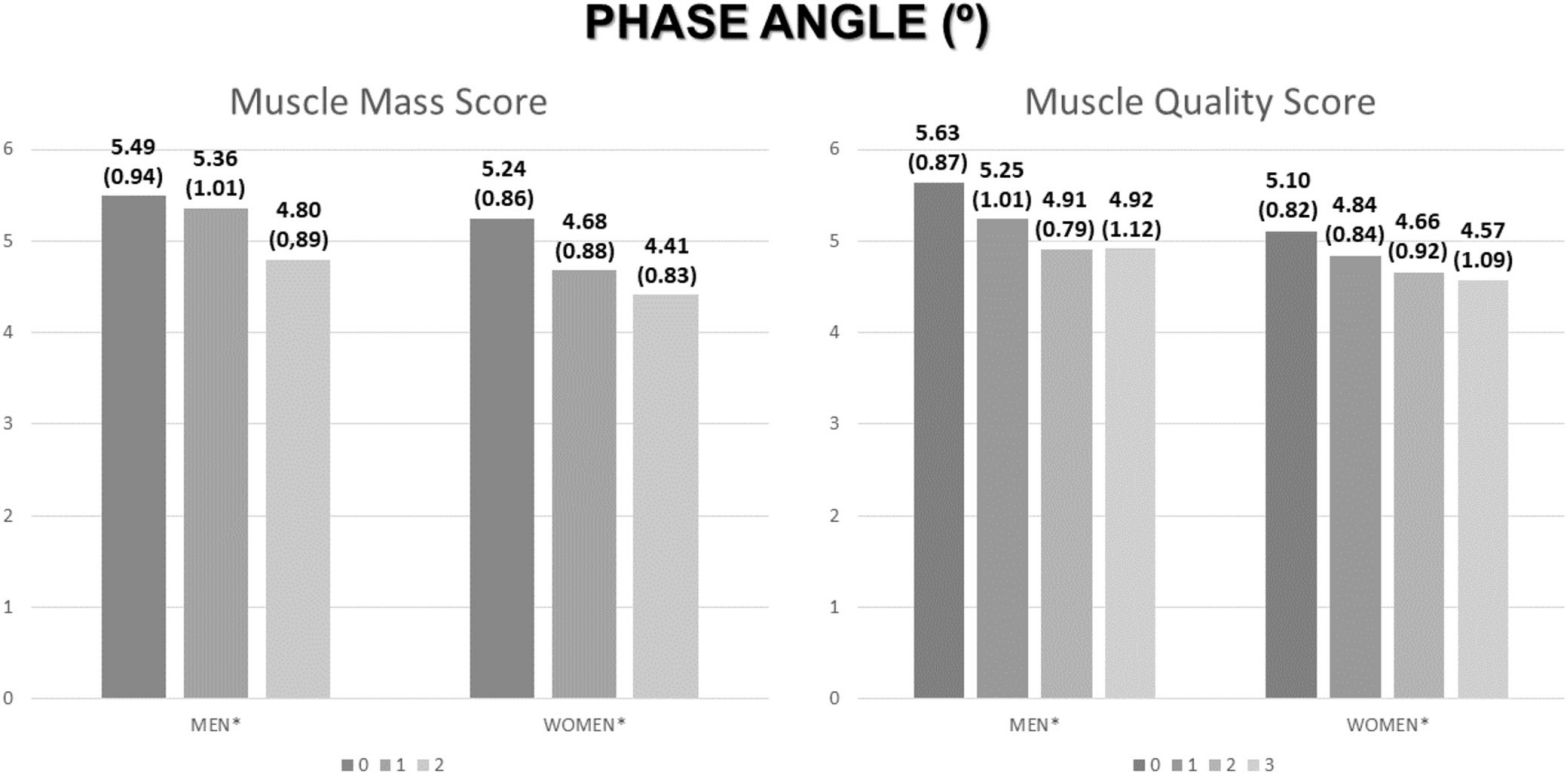
Differences in handgrip strength and phase angle related to muscle mass (RFMT + RFMA) and muscle quality score (MiT + FiT + Pennation Angle) in total sample. FATiT: medium echogenicity percentage; MiT: low echogenicity percentage; NMNFiT: high echogenicity percentage; RFMA: rectus femoris muscle area; RFMT: rectus femoris muscle thickness. Mean ± standard deviation.

**FIGURE 4:**
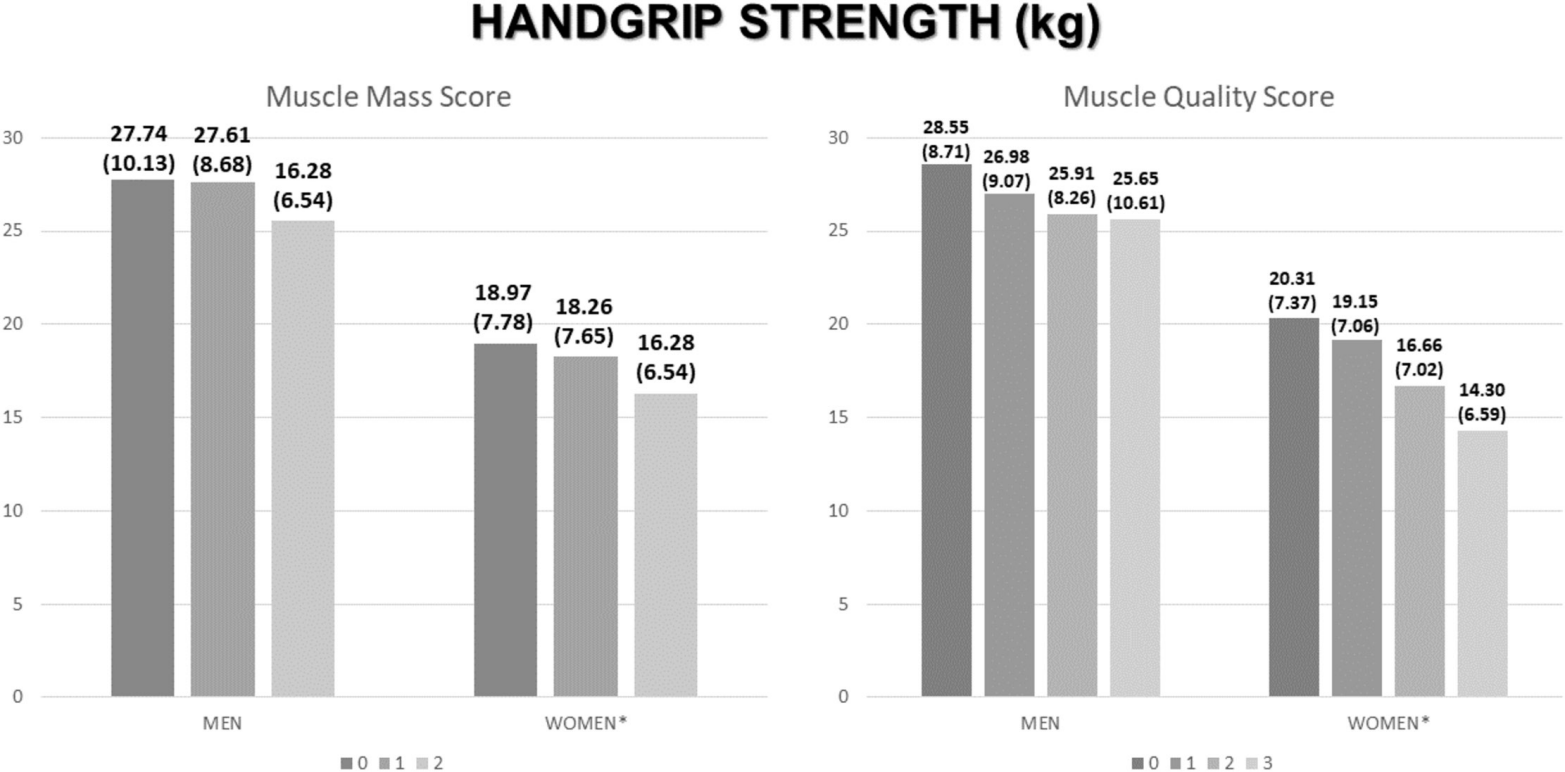
Differences in handgrip strength and phase angle related to muscle mass (RFMT + RFMA) and muscle quality score (MiT + FiT + Pennation Angle) stratified by sex. FATiT: medium echogenicity percentage; MiT: low echogenicity percentage; NMNFiT: high echogenicity percentage; RFMA: rectus femoris muscle area; RFMT: rectus femoris muscle thickness. Mean ± standard deviation *p < 0.05.

Patients with a higher score of muscle mass had lower phase angle (Figure 3) and lower handgrip strength (Figure 3). In the same way, patients with a higher score of muscle quality had lower phase angle (Figure 3) and lower handgrip strength (Figure 3). The differences were seen in aggregate, but in the stratification between sex, the differences in phase angle are maintained, but in handgrip strength, differences were only observed in women (Figure 4).

### 3.4 Relationship Between Muscle Mass and Quality With Morphofunctional Assessment

#### 3.4.1 Muscle Mass

There was a direct correlation between the variables of muscle mass determined by muscle ultrasonography (RFMA and RFMT) with phase angle and handgrip strength, and an inverse correlation between these muscle mass variables with resistance and reactance (Table 3).

**TABLE 3:** Correlations between phase angle, handgrip strength and ultrasonography variables.

	SFT (cm)	RFMA (cm^2^)	RFMT (cm)	Pennation angle (°)	MiT (%)	FATiT (%)	NMNFiT (%)
Phase angle (°)	*r* = −0.05; *p* = 0.19	*r* = 0.45; *p* < 0.01	*r* = 0.44; *p* < 0.01	*r* = 0.18; *p* < 0.01	*r* = 0.28; *p* < 0.01	*r* = − 0.21; *p* < 0.01	*r* = −0.29; *p* < 0.01
Resistance (ohm)	*r* = 0.17; *p* < 0.01	*r* = −0.32; *p* < 0.01	*r* = −0.24; *p* < 0.01	*r* = −0.12; *p* < 0.01	*r* = −0.03; *p* = 0.57	*r* = −0.02; *p* = 0.71	*r* = 0.02; *p* = 0.62
Reactance (ohm)	*r* = 0.19; *p* < 0.01	*r* = −0.31; *p* < 0.01	*r* = −0.23; *p* < 0.01	*r* = −0.11; *p* < 0.02	*r* = 0.01; *p* = 0.74	*r* = −0.04; *p* = 0.38	*r* = 0.08; *p* = 0.09
Handgrip strength (kg)	*r* = −0.35; *p* < 0.01	*r* = 0.38; *p* < 0.01	*r* = 0.34; *p* < 0.01	*r* = 0.18; *p* < 0.01	*r* = 0.21; *p* < 0.01	*r* = −0.17; *p* < 0.01	*r* = −0.21; *p* < 0.01

Abbreviations: FATiT: medium echogenicity percentage; MiT: low echogenicity percentage; NMNFiT: high echogenicity percentage; RFMA: rectus femoris muscle area; RFMT: rectus femoris muscle thickness; SFT: subcutaneous fat thickness.

#### 3.4.2 Muscle Quality

The pennation angle, as a muscle quality variable, showed a direct correlation with phase angle and handgrip strength, while it was inversely correlated with resistance and reactance (Table 3). AI‐assisted echogenicity variables also demonstrated specific correlations: the percentage of low echogenicity in the ROI exhibited a direct correlation with both phase angle and handgrip strength; the percentage of medium echogenicity in the ROI was directly correlated with phase angle but inversely correlated with handgrip strength; and the percentage of high echogenicity in the ROI showed an inverse correlation with both phase angle and handgrip strength (Table 3).

### 3.5 Relationship Between Parameters AI‐Assisted Muscular Ultrasonography With Sarcopenia and Dynapenia

To evaluate the relationship between the parameters obtained through AI‐assisted muscle ultrasound and the presence of sarcopenia and dynapenia, two models adjusted by sex, age and AI‐ultrasound variables were developed: one based on the absolute values of quantity and quality, and another using two scores generated according to the accumulation of pathological characteristics based on the median of the sample.

#### 3.5.1 Sarcopenia

Multivariate analysis by model I [age, sex and muscle mass quantitative variables (RFMT, RFMA and MiT)] showed male sex as a risk factor for sarcopenia, and higher muscle thickness as a protective factor for sarcopenia (Table 4).

**TABLE 4:** Model I: Risk factors for sarcopenia and dynapenia. Model II: Risk Factors for sarcopenia and dynapenia adjusted by age, sex, muscle score (mass and quality).

Model 1	OR	IC 95%	*p*‐value
Sarcopenia			
Age (years)	1.01	0.99–1.02	0.22
Gender (M/F)	1.85	1.23–2.77	< 0.01
RFMA (cm)	0.86	0.31–2.40	0.77
RFMT (cm)	0.18	0.04–0.86	0.03
MiT (%)	0.39	0.6–2.8	0.35
Dynapenia			
Age (years)	1.03	1.01–1.04	< 0.01
Gender (M/F)	1.40	0.97–2.03	0.07
RFMA (cm)	1.52	0.61–3.79	0.37
RFMT (cm)	0.33	0.08–1.30	0.11
MiT (%)	0.07	0.13–0.43	< 0.01
Model 2	OR	IC 95%	*p*‐value
Sarcopenia			
Age (years)	1.01	1–1.03	< 0.05
Gender (M/F)	1.29	0.89–1.86	0.18
Muscle mass score	1.51	1.22–1.87	< 0.01
Muscle quality score	1.22	1.016–1.47	0.03
Dynapenia			
Age (years)	1.03	1.02–1.05	< 0.01
Gender (M/F)	1.13	0.81–1.57	0.49
Muscle mass score	1.08	0.89–1.29	0.43
Muscle quality score	1.35	1.14–1.59	< 0.01

Abbreviations: F: female; FATiT: medium echogenicity percentage; M: male; MiT: low echogenicity percentage; NMNFiT: high echogenicity percentage; RFMA: rectus femoris muscle area; RFMT: rectus femoris muscle thickness.

Multivariate analysis by model II showed a higher muscle quality score (worse muscle quality) as a risk factor for sarcopenia and a higher muscle mass score (low muscle mass) was a risk factor only for sarcopenia (Table 4).

#### 3.5.2 Dynapenia

Multivariate analysis by model I for dynapenia showed that ROI low echogenicity percentage (MiT) was a protective factor (Table 4).

Multivariate analysis by model II showed a higher muscle quality score (worse muscle quality) as a risk factor for dynapenia (Table 4).

## 4 Discussion

This study aims to evaluate the relationship between a functional muscle disease, such as sarcopenia and ultrasonographic features obtained through an AI tool applied to clinical practice images. The main findings revealed that muscle mass characteristics, such as muscle area and muscle thickness of the quadriceps rectus femoris, showed a direct correlation with handgrip strength and phase angle in patients at risk of malnutrition. Muscle quality parameters, such as the low echogenicity percentage from the region of interest (MiT), demonstrated a weak direct correlation with these functional parameters, while medium echogenicity percentage (FiT) and high echogenicity percentage (NMNFiT) exhibited a weak inverse correlation with functional measures like handgrip strength and phase angle. When considering muscle mass and muscle quality as two separate scores derived from ultrasound imaging characteristics, patients with poorer scores (higher values) were found to have an increased risk of sarcopenia and dynapenia, even after adjustments for gender and age.

Muscular ultrasonography has emerged as a valuable technique for evaluating muscle status in patients due to its ease of implementation in clinical and bedside settings. While several muscle groups can be assessed, the rectus femoris of the quadriceps is among the most accessible and has substantial evidence supporting its use for prognosis and monitoring across various patient populations. Perkisas et al. proposed five parameters for muscle assessment: thickness, cross‐sectional area, echogenicity, fascicle length and pennation angle [25]. In our study, we evaluated four of these parameters with the assistance of an AI tool for automatic segmentation, which also enabled the assessment of muscle quality based on echogenicity through an algorithm that differentiates muscle mass, fat mass and other structures from ultrasound images.

Baseline patient data revealed differences between men and women in both muscle mass and muscle quality, with men showing greater muscle area, thickness, pennation angle and muscle percentage in the region of interest (ROI). These findings align with previous evidence: Stausholm et al. reported greater muscle thickness in healthy men [26]; and the DRECO study observed increased muscle area in men with disease‐related malnutrition [3]. Differences in muscle percentage and echogenicity have also been noted in studies of patients with disease‐related malnutrition [12] and neurologic diseases [9]. These results are consistent with other previously reported differences in functional parameters, such as phase angle and handgrip strength, as reported in the literature [2, 27].

Muscle mass parameters are lower in patients with sarcopenia compared to those without sarcopenia. These findings align with existing evidence, as sarcopenia is defined by a reduction in muscle mass and function. Several studies, such as the DRECO study conducted in patients with disease‐related malnutrition [3] and the meta‐analysis by Yang et al. focusing on adults aged > 65 years [28], have observed reductions in muscle mass using ultrasonography. However, muscle quality is less thoroughly assessed in patients with sarcopenia, and defining this altered muscle condition remains challenging when aiming to understand the true impact of disease and aging on muscles. In our sample, patients with sarcopenia exhibited lower pennation angles and low echogenicity percentage in the region of interest (ROI), assumed as muscle percentage, but no significant differences in medium echogenicity percentage, assumed as fat, were observed, indicating no notable differences in fat infiltration between the groups. These findings could be linked to the cut‐off points for low muscle mass in sarcopenia proposed by EWGSOP2 for BIA (< 7 kg/m^2^ for men and < 5.5 kg/m^2^ for women) [2], which are validated for older adults [2]. This is further supported by the observed differences in women but not in men.

Furthermore, when analysing muscle quality parameters in relation to dynapenia, significant differences were found across all parameters, including MiT, FiT, NMNFiT and pennation angle. Assuming that low echogenicity reflects muscle mass, medium echogenicity reflects fat mass, and high echogenicity reflects other tissues, these results suggest that muscle mass loss does not always directly correspond to sarcopenia. Alterations in muscle architecture, including a decrease in the number of muscle fibres, reduced fibre diversity and changes in fibre type composition, may precede the onset of sarcopenia and impact muscle strength. These findings are consistent with those reported in previous research [29].

Phase angle and muscle strength, as indicators of muscle mass and function, showed moderate correlations with muscle mass parameters (thickness and area) measured by ultrasonography. The lack of a strong correlation may be attributed to the heterogeneous nature of the sample. Previous studies on patients with disease‐related malnutrition, with similar distributions, have reported comparable correlations [30] Additionally, a study by Zhao et al. observed low to moderate correlations between muscle mass assessed by BIA or DXA and parameters such as cross‐sectional area and muscle thickness [31]. Muscle quality parameters demonstrated low but significant correlations with phase angle and handgrip strength—direct correlations with MiT and pennation angle, and inverse correlations with FiT and NMNFi. Echogenicity of muscle, as measured by ultrasonography, is influenced by the presence of fibrous and adipose tissues, which are associated with decreased phase angle and impaired muscle function [32]; Furthermore, echogenicity correlates with myosteatosis, as evidenced by findings from the study conducted by Akima et al. [33].

Multivariate analysis identified an association between muscle thickness and the risk of sarcopenia assessed by BIA, as well as between MiT percentage and the risk of dynapenia. These findings may be influenced by the limitations of the technique used to diagnose these conditions, as ultrasound imaging can be affected by factors such as hydration, intra/extracellular balance, muscle damage and the absence of muscle atrophy in the early stages of altered muscle function [26, 34]. The use of qualitative scores, based on the sample median, for muscle mass and quality allowed us to integrate key parameters, including muscle size and muscle quality derived from AI‐generated data. These scores revealed an increased risk of sarcopenia associated with altered muscle mass and quality scores. However, the risk of impaired muscle strength was linked solely to the muscle quality score, which assigned higher points to reductions in Mi and pennation angle, alongside increases in FATi. Isaka et al. reported that combining ultrasonography parameters with appendicular skeletal muscle mass index and handgrip strength, though their index incorporated both muscle mass and functional parameters and focused on other muscle groups [35]. The findings from the proposed muscle scores suggest the presence of muscle quality alterations affecting function prior to the onset of muscle atrophy. AI‐based ultrasound offers potential biomarkers for assessing muscle health and monitoring the impact of medical interventions in these patients.

The main strengths of this study include the validation of tools to assess muscle quality in a large cohort of patients with sarcopenia. Muscle quality is a key feature of sarcopenia, yet it is challenging to evaluate in clinical practice. Additionally, the observed correlations between muscle mass, muscle quality parameters and their combined scores with sarcopenia further support the use of muscular ultrasonography in routine practice.

The study's main limitations are the use of a heterogeneous sample comprising patients with various causes of disease‐related malnutrition. This variability may hinder the ability to identify strong correlations between parameters. Furthermore, due to the absence of established cut‐off points for muscle quality in sarcopenia, we used a variable based on the median of the sample to evaluate it. Although in some cases the full cross‐sectional area of the rectus femoris muscle could not be captured due to the limited field of view, the analysis was based on the visible area within the image. This may have influenced the generalizability of RFMA values, particularly in individuals with greater muscle mass but the differences between those with sarcopenia and those who are not are significant. However, muscle thickness, an independent and clinically meaningful parameter, was consistently assessed and provides a robust measure of muscle status across participants. Another technical limitation is the thresholding of muscle quality analysis for muscle, fat and no muscle no fat indices that are highly dependent on several factors, including the ultrasound scanner and patient characteristics, such as fat thickness; the standardized settings of ultrasound images were chosen to ensure consistency in image acquisition and to reduce the influence of technical variability on the histogram‐based analysis. While anatomical differences such as fat thickness may still affect image characteristics, the use of a uniform scanning protocol and trained personnel helped mitigate these effects. While echogenicity‐based classification has limitations, the use of a validated segmentation algorithm combined with consistent imaging conditions provides a reliable framework for distinguishing tissue types. However, the method could gain further credibility through validation against biological tissue samples. That said, such validation is challenging to implement due to ethical constraints. On the other hand, the study design does not allow for causal inferences or longitudinal assessment of changes in muscle mass and quality over time. Although handgrip strength and phase angle were evaluated, other functional performance tests (e.g., gait speed and chair stand test) were not included, potentially limiting the functional interpretation of sarcopenia diagnosis. Similarly, the chair stand test is considered the most appropriate method for evaluating quadriceps strength and its relationship with quadriceps ultrasound measurements. However, since this study involved ultrasound assessment of the quadriceps conducted in a real‐world clinical setting, we were unable to perform this test due to time constraints during patient consultation. Finally, the study was conducted in a single institution, which may limit the generalizability of the findings to other populations or healthcare settings.

The future lines of investigation that may emerge from this study are related to the understanding of muscle quality and its relationship with muscle function. Further validation studies are necessary to implement these techniques in routine clinical practice. Additionally, future validation studies could be conducted using other diagnostic tools that may serve as gold standards. On the other hand, the AI muscle quality features studied in this study can be evaluated in the prognosis of patients with diseases related to malnutrition and sarcopenia. Additionally, the use of scores that jointly evaluate various variables related to muscle mass and quality can facilitate the creation of clusters to assess different clinical features, enabling more precise medical nutrition therapy.

## 5 Conclusions

AI‐based muscle ultrasound imaging system plays a potential role in evaluating muscle mass and quality in patients with disease‐related malnutrition. Muscle mass characteristics showed direct correlations with handgrip strength and phase angle, while muscle quality parameters exhibited weaker but significant correlations. The development of muscle mass and quality scores based on AI‐assisted ultrasonography indicated an increased risk of sarcopenia assessed by BIA, while muscle quality scores alone were associated with a risk of reduced muscle strength (dynapenia), adjusted for age and sex.

AI‐based ultrasound has emerged as a valuable tool for identifying biomarkers of muscle health and monitoring therapeutic interventions. This study highlights the potential of integrating AI tools into routine clinical practice for earlier and more precise diagnosis and management of sarcopenia and related conditions. Future research should focus on validating these techniques and exploring their prognostic applications.

## Ethics Statement

The study was approved by the Medical Research Ethics Committee (CEIm) of the East Valladolid Area (code: PI 20‐1886 and PI 23‐341). All study procedures complied with the principles of the Declaration of Helsinki. Signed informed consent was obtained from all eligible participants prior to enrollment.

## Conflicts of Interest

A.C. and E.J.G. were employed by DAWAKO Medtech SL. The other authors declare no conflicts of interest.
